# Novel definition files for human GeneChips based on GeneAnnot

**DOI:** 10.1186/1471-2105-8-446

**Published:** 2007-11-15

**Authors:** Francesco Ferrari, Stefania Bortoluzzi, Alessandro Coppe, Alexandra Sirota, Marilyn Safran, Michael Shmoish, Sergio Ferrari, Doron Lancet, Gian Antonio Danieli, Silvio Bicciato

**Affiliations:** 1 Department of Biomedical Sciences, University of Modena and Reggio Emilia, via G. Campi 287, 41100, Modena, Italy; 2 Department of Biology, University of Padova, via G. Colombo 3, 35131, Padova, Italy; 3 Department of Molecular Genetics, The Weizmann Institute of Science, Rehovot 76100, Israel; 4 Department of Biological Services, The Weizmann Institute of Science, Rehovot 76100, Israel; 5 Bioinformatics Knowledge Unit, The Lorry I. Lokey Interdisciplinary Center for Life Sciences and Engineering Technion, Israel Institute of Technology, Haifa, Israel; 6 Department of Chemical Engineering Processes, University of Padova, via F. Marzolo 9, 35131, Padova, Italy

## Abstract

**Background:**

Improvements in genome sequence annotation revealed discrepancies in the original probeset/gene assignment in Affymetrix microarray and the existence of differences between annotations and effective alignments of probes and transcription products. In the current generation of Affymetrix human GeneChips, most probesets include probes matching transcripts from more than one gene and probes which do not match any transcribed sequence.

**Results:**

We developed a novel set of custom Chip Definition Files (CDF) and the corresponding Bioconductor libraries for Affymetrix human GeneChips, based on the information contained in the GeneAnnot database. GeneAnnot-based CDFs are composed of unique custom-probesets, including only probes matching a single gene.

**Conclusion:**

GeneAnnot-based custom CDFs solve the problem of a reliable reconstruction of expression levels and eliminate the existence of more than one probeset per gene, which often leads to discordant expression signals for the same transcript when gene differential expression is the focus of the analysis. GeneAnnot CDFs are freely distributed and fully compliant with Affymetrix standards and all available software for gene expression analysis. The CDF libraries are available from , along with supplementary information (CDF libraries, installation guidelines and R code, CDF statistics, and analysis results).

## Background

Affymetrix technology is widely used for the analysis of transcriptional profiles and most gene expression data available in public repositories have been produced using different generations of Affymetrix GeneChips. In this type of microarrays, the expression signal of each transcript is quantified summarizing the intensities of all the oligonucleotides, i.e. the probes (e.g., 11 or 16), of a probeset matching a target gene or transcript. The signal can be generated using a series of statistical or model-based algorithms (i.e., MAS5.0, MBEI, RMA, GCRMA, PLIER, PDNN). Despite the computational differences, all methods for signal quantification rely on the correspondence between probes and genomic sequences. The Affymetrix Chip Definition Files (CDFs) encode the physical design of the microarray and contain the sequence details to link the oligonucleotide probes of the chip to the interrogated transcripts. The information of a CDF file relies so deeply on the genome annotation contained in the databases that the same name of the chip reflects the version of the UniGene Build used for probe design (e.g., the HG-U133 expression set and the human UniGene Build 133). The evolution of genome sequence annotation from the time when probesets were designed caused a massive deviation from the original one-to-one probeset/transcription locus (i.e. UniGene entry) assignment. Several studies revealed the existence of a considerable gap in the correspondence between Affymetrix probes and probesets with genes and transcripts [1-8]. Affymetrix continuously updates probesets annotations and redefines the links between probesets and genes indicating the UniGene cluster that contains the probeset representative sequences and linking them to the corresponding EntrezGene ID. Similarly, the Bioconductor Biocore team quarterly releases CDFs and annotation libraries at the Bioconductor website, which can be used for analysis of gene expression data in R environment. However, these update actions simply affect the qualitative attributes of probesets without any degree of control on the effective matching between probes and genome sequences. As such, Dai et al. [6] developed a novel system for associating probes to genomic information, based on custom-probesets which are composed of at least four probes specifically matching the same sequence. Dai and coworkers defined custom-probesets based on updated versions of RefSeq, EntrezGene as well as ENSEMBL Gene, Transcript and Exon entries and generated custom CDFs for the most popular Affymetrix GeneChips [9]. The development of custom CDF was shown to deeply improve the analysis outcome when the focus of the experiment is the identification of differentially expressed genes [5,6]. Furthermore, the assembly of Dai et al., based on different sources of information, provides a set of custom CDFs useful for different analytical purposes. Nevertheless, in these CDFs a specific probe may be included in more than one custom-probeset, thus introducing some uncertainty in the association between probe signal and overall expression level of corresponding transcripts. As an example, in version 8 of the RefSeq-based set of human probesets for the HG-U133A array, 26% of probes are included in two or more probesets and 48% of probesets share probes with other probesets.

More recently, Lu et al. [7] developed custom-probesets definitions for Affymetrix GeneChips based on transcript sequences from the AceView database. Custom-probesets defined by Lu et al. are reorganized groups of probes, specifically matching the same transcript or the same group of transcript sequences, independently from their original inclusion in different Affymetrix probesets. Lu et al. showed that their probesets are able to discriminate between differential expressions of specific transcript variants. Although addressing the issue of multiple transcript variants, this approach still present the limitation that most of the redefined transcript-related probesets match more than one transcript, thus hampering the discrimination between differential expression of a specific transcript variant. In this work we explore the association between probesets and genes and transcripts and define alternative Chip Definition Files for Affymetrix 3' expression arrays with the intent to reduce the impact in signal quantification of probes matching more than one gene and/or of probes which do not match any transcribed gene. Although there is a growing interest in using microarray platforms to detect events related to the complexity of gene structure, e.g., multiple transcripts per gene, alternative splicing and exon differential expression, the identification of differentially expressed genes is still the major goal of microarray-based expression studies and 3' expression arrays still represent the most abundant source of data contained in public repositories. The use of GeneAnnot CDFs (GA_CDFs) is intended to improve gene-centered analysis of transcriptional data where the focus is in the reliable identification of genes, rather than individual transcripts, that are differentially expressed. Other aspects related to individual transcript variants, alternative splicing and exon differential expression, although, in principle, detectable with 3' expression arrays, can be more efficiently investigated using dedicated technologies such as Affymetrix genome-wide, whole-transcript coverage arrays.

Our set of custom CDFs and corresponding Bioconductor packages (i.e. CDF, probe and annotation libraries) for Affymetrix human gene chips are based on the GeneAnnot database which contains the comparison of any Affymetrix probe with transcript sequences from publicly available cDNAs, GenBank, RefSeq and Ensembl repositories [10].

## Implementation

GeneAnnot was created as part of the GeneCards human gene indexing database [11] to explore the many-to-many relationships between probesets and genes. GeneCards hierarchically defines a gene based on three major sources, the HUGO gene nomenclature committee (HGNC) database [12], Entrez Gene, and Ensembl. Every gene present in the first source obtains a HGNC symbol, and is clearly linked to the other two sources. Other genes obtain their symbol from the other two sources. As such, GeneCards has an inclusive list of genes from all three sources, with extensive mutual links and connections to more than 50 databases. In GeneAnnot, each probe from Affymetrix probesets is matched with transcript sequences from GenBank, RefSeq and Ensembl databases, and then transcripts are linked to GeneCards genes [10].

The novel set of custom GeneChip CDFs, named GA_CDFs, and the corresponding Bioconductor probe and annotation libraries, have been designed using GeneAnnot and GeneCards. GA_CDF files are currently available for the human GeneChips HG-U95 set, HG-U133 set and HG-U133 Plus 2.0, based on GeneAnnot version 1.4a, synchronized with GeneCards Version 2.35.

GA_CDF have been designed using the concept of gene-related custom-probesets, starting from the subset of Affymetrix GeneChip probes that matches transcripts specifically linked to a single GeneCards gene. Probes have been first aggregated into putative custom-probesets, each one including only those probes with a unique and exclusive correspondence with a single GeneCardsID. Probe to sequence correspondence has been quantified allowing a single mismatch in the comparison between the Affymetrix 25-mer and the target sequence [10]. Then, custom-probesets have been retained and included in the custom CDF if, and only if, they contained at least 11 probes (GA11_CDF), i.e. the minimum number of probes in standard Affymetrix probesets. To evaluate the impact of the number of probes making up a custom-probeset, all the analyses have been also performed using an alternative CDF, GA6_CDF, composed of custom-probesets including a minimum of 6 probes, selected with the same criteria adopted for GA11_CDF. Probeset names were generated adding the suffix "_at" to the corresponding GeneCardsIDs. Custom CDF, probe and annotation Bioconductor libraries, fully compliant with Affymetrix standards, have been constructed using dedicated functions based on R and Bioconductor packages. As such, Bioconductor users can easily take advantage of these libraries, e.g., replacing, in AffyBatch objects, the values of "cdfName" and "annotation" slots (supplementary information). Moreover, GeneAnnot CDF can also be used with all third-party software adopting Affymetrix standards, e.g., dChip.

## Results

The quality of GeneAnnot custom CDFs was tested and compared with other CDF files on the same experiment used by Dai and co-workers to assess the impact of probeset definition on the differential expression. The data set is available at Gene Expression Omnibus GSE974 and consists of paired HG-U133A arrays hybridized with RNA from the heart tissue of 19 patients with heart failure. Samples were obtained at the implant and then at the explant of a left ventricular assistant device. We compared results obtained applying a standard analytical approach on gene expression data generated using six different CDF packages, specifically i) Bio_CDF, the Biocore hgu133acdf library obtained from [13]; ii) Entrez8_CDF, based on EntrezGene database and iii) RefSeq8_CDF, based on RefSeq (CDF version 8 from Dai et al. [6], available at [9]); iv) AV_CDF, based on AceView database (CDF version 1.12.0 from Lu et al. [7], available at [14]); v) GA6_CDF and vi) GA11_CDF, the custom CDFs derived from GeneAnnot and containing meta-probesets composed of at least 6 and 11 probes per gene, respectively [15].

Probeset level data was generated through RMA with default parameters and analyzed using SAM with the paired data method in the R environment. According to the procedure adopted by Dai et al. [6], lists of differentially expressed genes (DEG) showing at least a 20% change (i.e., fold change of at least 1.2) were generated at SAM q-value thresholds of 1, 5, and 10%. Results obtained using the various CDF files at the different q-value thresholds are reported in Table 1. Regardless of chip definition file and q-value cut-offs, the total number of DEG is quite stable when utilizing different CDFs, especially at more stringent thresholds, and normally from 20 to 40% of DEG identified using Bio_CDF are not included in DEG obtained with custom CDFs. Similarly, pair-wise comparisons of the differentially expressed genes obtained with the various CDFs indicate that about 30% of the DEGs identified using a CDF cannot be confirmed by the analysis based on another definition file. This evidence is in accordance with the result from Dai et al. [6] that, on average, about 40% DEG found with a CDF cannot be confirmed by the others. The pair-wise comparisons among Entrez8_CDF andRefSeq8_CDF presented by Dai et al. [6] and GeneAnnot CDFs indicate that the two approaches produce definition files which generate the most comparable results (on average 82% of commonly identified DEG).

**Table 1:** Differentially expressed genes selected using different CDFs.

**CDF type**	**Number of DEG**	**DEG found by all CDFs**		**DEG shared in the pair-wise comparisons of lists generated using different CDFs**					
								
		Number	%						
**q-value < 1%**									
Bio	42	17	40.5		Entrez8	RefSeq8	AceView	GA6	GA11
Entrez8	28		60.7	Bio	25	29	22	33	33
RefSeq8	34		50	Entrez8		25	20	28	28
AceView	35		48.6	RefSeq8			22	29	29
GA6	46		37	AceView				23	23
GA11	42		40.5	GA6					42
**q-value < 5%**									
Bio	76	42	55.3		Entrez8	RefSeq8	AceView	GA6	GA11
Entrez8	99		42.4	Bio	51	54	52	56	54
RefSeq8	88		47.7	Entrez8		82	64	92	86
AceView	92		45.7	RefSeq8			63	82	77
GA6	109		38.5	AceView				66	63
GA11	99		42.4	GA6					99
**q-value < 10%**									
Bio	140	73	52.1		Entrez8	RefSeq8	AceView	GA6	GA11
Entrez8	125		58.4	Bio	89	94	97	100	96
RefSeq8	125		58.4	Entrez8		112	90	111	106
AceView	129		56.6	RefSeq8			94	109	103
GA6	139		52.5	AceView				93	89
GA11	130		56.2	GA6					129

Results from samr analysis of GSE974 dataset using six different CDFs under different q-value cut-offs in terms of number of total identified differentially expressed genes (DEG), of number and percentage of DEG in common by all lists generated using the various CDFs, and of number of DEG present in the pair-wise comparison of lists generated using different CDFs. EntrezGene IDs were used as reference identifiers to verify overlap of lists.

## Discussion

A novel set of custom CDF and the corresponding Bioconductor libraries for Affymetrix human 3' expression arrays has been developed based on GeneAnnot and GeneCards information. GeneCards is a popular and widely used database integrating gene-centered information from major databases, which could show some inconsistencies among themselves if considered singularly. GeneAnnot based CDFs are provided with libraries compliant with Bioconductor standards, including probe libraries that are required for sequence level analysis, such as GCRMA pre-processing, and annotation libraries that take advantage of the rich annotations that are reported in GeneCards, thus facilitating their implementation by final users.

GeneAnnot custom CDFs address the problem of a reliable reconstruction of expression signals through the inclusion in a unique custom-probeset of only those probes matching transcripts associated to a single gene. Different groups in recent years have proposed a variety of methods to re-define probesets from Affymetrix 3' arrays referring to several databases and proposing different strategies to solve the technical issues of probeset composition and matching [6,7]. Each approach has advantages and disadvantages, although the definition of custom-probesets has little effect on the general performance and the results of sample clustering and classification [7,16]. Thus, the decision on which is the most appropriate custom-CDF to be used, largely depends on the goals of the experimenter: if the major purpose of the study is to analyze samples based on the expression patterns, using either probeset definition leads to similar results. When the focus comes down to the identification of specific genes, then the most appropriate CDF has to be carefully selected considering issues related to the database and the strategy used to group probe pairs into custom-probesets. Specifically, if the biological relevancy is in detecting differentially expressed genes, using custom CDFs which refer to *gene-centered database *and combine all probes per gene into a single probeset may be the best choice. On the other hand, if the focus is distinguishing expression of individual transcript variants, then relying on a *transcript-centered database *and sub-dividing probesets into small groups of probes (e.g., 4–5 probes) covering individual exons may be a more appropriate approach. GeneAnnot and GA_CDFs have been proposed for improving the reliability of results from *gene-centered *analysis of microarray experiments. In this regards, they aimed at eliminating the presence of more than one probeset per gene, a frequent instance in Affymetrix standard probeset definitions which often leads to discordant expression signals when the focus of the analysis is detecting differentially expressed genes. Consequently, GA_CDFs may not represent the CDFs of choice when 3' expression arrays are used to detect transcript variants, alternative splicing and exon differential expression.

Using different CDFs directly reflects on different utilization of the probe-level information available in the chip for signal reconstruction. Expression data generated using the Bio_CDF are based on all probes (100%) contained in the chip while data obtained with the Entrez8_CDF rely on the 68.7%. Similarly, AV_CDF accounts for 77.3% of all probes when considering probesets with at least 4 probes [7]. In the latter case, indeed, 48% of probesets have less than 4 probes and therefore may not support reliable statistical summarization, as assessed by Lu et coworkers [7]. Consequently, undesired probesets with less than 4 probes should be filtered out before pre-processing procedures, such as RMA, and this filtering could be not trivial for final users. GA11_CDF has been constructed using more than 77.5% of the probe-level information and all custom-probesets include at least 11 probes, i.e. the minimum number of probes in the standard Affymetrix probesets. As discussed in [6] and [7], a probeset composed of at least four probe pairs should satisfy the minimum requirements of most probe-level analysis algorithms and, thus, any choice on the number of probes composing a custom-probeset is largely arbitrary. We imposed that each custom-probeset be represented by at least 11 probes because in our GeneAnnot-based probesets definition 90% of GeneCardsIDs are interrogated by custom-probesets composed of at least 11 probe pairs (supplementary information). Nevertheless, the functions to create the custom-probesets are generally applicable and the minimum number of probe pairs making up a custom-probeset is a tunable parameter. A comparison between the lists of differentially expressed genes obtained using GA11_CDF and a GeneAnnot custom CDF with probesets composed of 6 probe pairs (GA6_CDF, accounting for 95% of GeneCardsIDs) indicates that the impact of this parameter is minimal (Table 1).

The various CDFs result in different number of genes whose transcripts levels are measured by a probeset or by groups of probesets. In particular, the Bio_CDF contains probesets associated to 13,389 EntrezGenes, while the Entrez8_CDF accounts for 11,999 EntrezGenes. GA6_CDF and GA11_CDF custom-probesets are annotated to 12,074 and 11,408 EntrezGenes respectively, thus representing comparable number of annotated genes when compared with other gene-centered custom CDFs.

In addition, the deviation from the one-to-one probeset/gene match is variable in the different definition files, due both to the existence of multiple probesets per gene (or multiple probesets per transcript as in AV_CDF) [8] or to the presence of the same probe in multiple custom-probesets, that adds ambiguity in the evaluation of signals (e.g. in the RefSeq8_CDF probes with indexes 182067, 182068, 204881, and 204883 are present in 39 different custom-probesets). On the contrary, GeneAnnot based custom-probesets include only probes matching transcripts linked to a single gene. As such, they preserve a one-to-one correspondence between genes and custom-probesets. Furthermore, each probe is assigned to a unique custom-probeset, thus avoiding additional noise due to the use of a probe into multiple probesets.

Finally, the reported data show that, when applied to the analysis of a standard experimental design, GA_CDFs perform similarly to the other custom CDFs, with the additional advantage that GeneAnnot based CDFs are provided with complete annotation libraries compliant with Bioconductor standards, thus allowing an easier implementation by final users.

## Conclusion

This work present a novel set of custom CDFs for Affymetrix human GeneChips, based on GeneAnnot and GeneCards. Although other alternative CDFs have been recently released, GeneAnnot based custom CDFs constitute a valuable alternative to Affymetrix and custom Chip Definition Files since i) they are based on GeneCards, an extensively-used database integrating information from different sources; ii) address the problem of multiple probesets per gene as well as the problem of probes matching different genes within the same probeset; iii) exploit an high percentage of the GeneChips probes, and iv) could be easily adopted by final user since they are provided with Bioconductor-compliant libraries, including probe and annotations libraries, that will be continuously updated according with novel GeneAnnot and GeneCards releases.

## Availability and requirements

**Project name: **GeneAnnot custom CDF files

**Project home pages: ** and 

**Operating systems: **platform independent

**Programming language: **R scripting language

**Other requirements: **R statistical environment 2.4 or higher and Bioconconductor 1.9 or higher are required to use Bioconductor-compliant packages. Standalone CDF files can be used with any software adopting Affymetrix standards for CDF files.

**License: **GPL

## Authors' contributions

FF wrote the code for custom CDF reconstruction and carried out the comparison analyses; SBi and SBo conceived the study and carried out the comparison analyses; AC and AS participated to the processing and integration of original information from public databases; MSa, MSh, SF, DL and GAD supervised the work. FF, SBi and SBo wrote the manuscript, which was revised by all Authors.
